# Role of Vagus Nerve Stimulation in Refractory and Super Refractory Status Epilepticus: A Pediatric Case Series

**DOI:** 10.3390/brainsci13111589

**Published:** 2023-11-14

**Authors:** Giulia Melinda Furlanis, Jacopo Favaro, Nicola Bresolin, Daniele Grioni, Valentina Baro, Alberto D’Amico, Stefano Sartori, Luca Denaro, Andrea Landi

**Affiliations:** 1 Pediatric and Functional Neurosurgery, Department of Neuroscience, Padua University Hospital, via Giustiniani 5, 35127 Padova, Italy; giuliamelinda.furlanis@aopd.veneto.it (G.M.F.); andrea.landi@unipd.it (A.L.); 2 Neurology and Neurophysiology Unit, Department of Women’s and Children’s Health, Padua University Hospital, 35128 Padova, Italy; jacopo.favaro@aopd.veneto.it (J.F.);; 3 Epilepsy Unit, Villa Santa Maria SCS, 22038 Como, Italy

**Keywords:** super refractory status epilepticus, vagus nerve stimulation, neuromodulation, pediatric epilepsy, NORSE, FIRES

## Abstract

Background: Status epilepticus is a life-threatening condition that is defined as refractory (RSE) when the seizure activity continues despite treatment with benzodiazepine and a second appropriate treatment. Super refractory status epilepticus (SRSE) is an RSE that persists or recurs for ≥24 h. Few papers have reported the outcomes of pediatric patients affected by RSE and SRSE and treated with neuromodulation therapies. Vagus nerve stimulation (VNS) is an approved treatment for drug-resistant epilepsy. We present our findings of pediatric patients treated with VNS for RSE/SRSE. Methods: We present a case series of seven consecutive pediatric patients treated with VNS for SRSE since 2012 by a single surgeon in Monza and Padua. A rapid titration was started soon after implantation. We considered electroclinical data before and after VNS implantation and at the last follow-up. Results: We achieved the resolution of SRSE in five out of seven patients in a mean time of two weeks. At the last follow-up, these patients had a significant reduction of seizure burden without any relapse of SE. Discussion and Conclusions: Based on our limited findings, we discuss the potential role of VNS therapy in similar but distinct clinical contexts. For patients with drug-resistant epilepsy and RSE/SRSE, prompt VNS consideration is suggested, offering rapid responses and potentially reducing pharmacological load. Meanwhile, in NORSE/FIRES, we suggest early neuromodulation during the acute phase if standard treatments prove ineffective or not tolerated. This approach may leverage VNS’s potential anti-inflammatory effects and neuromodulation, enhancing patient-specific treatments. Expanding case studies and prolonged follow-ups are recommended to strengthen these clinical insights.

## 1. Introduction

Status epilepticus (SE) is a life-threatening medical emergency defined as “a condition resulting either from the failure of the mechanisms responsible for seizure termination or from the initiation of mechanisms which lead to abnormally prolonged seizures (after time point t1)”. This t1 operates as follows: 5 min for tonic-clonic SE and 10 min for focal SE with impaired consciousness. SE is defined as refractory (RSE) when ictal activity continues despite treatment with benzodiazepine and a second appropriate anti-seizure medication (ASM) [1,2]. In children, the second ASM appears to be far less effective than the first. RSE develops in 23–43% of patients with SE and leads to a return to a baseline neurological status in a few cases. RSE leads to death in 17–39% of adults, whilst lower rates are found in children [3]. Super refractory status epilepticus (SRSE) is an RSE that persists or recurs for ≥24 h despite treatment with an anesthetizing ASM [4]. SRSE bears a high risk of poor neurological outcome, progressive brain atrophy, prolonged hospitalization, intensive unit-level care, and mortality. 

Little is still known about the natural history of RSE and SRSE in pediatric patients, especially in the context of NORSE/FIRES (new onset refractory status epilepticus/febrile infection-related epilepsy syndrome), which is recognized as a rare disorder. Estimating the exact incidence and mortality of SRSE is challenging, but a large retrospective study showed an incidence of 7.14% among convulsive SE in children [5]. The nature of RSE/SRSE is widely heterogeneous, and prognosis depends highly on the etiology and duration of SE, with a mortality rate of 13.7–43.5% [6]. Acute symptomatic or progressive encephalopathy groups, as well as prolonged and refractory cases, have a worse prognosis. Most children will continue to have seizures, and unlike the situation in adults, a seizure-free state is ultimately unlikely [7]. 

The annual incidence of FIRES among children and adolescents is estimated to be 1:1,000,000, according to the German nationwide hospital-based prospective surveillance program. According to the literature, children with NORSE experience hundreds of seizures per day during the acute phase, which lasts from a few days to several months. This first acute phase is typically followed by a second chronic phase with refractory epilepsy, neurological and cognitive impairment, and functional disability. The mortality rate is around 12% in children, and a vegetative state is not rare [8]. 

To the best of our knowledge, there are no guidelines to treat RSE and SRSE; the treatment is heterogeneous, based mainly on expert opinions, and lacks controlled clinical studies. The role of dietary, immunological, surgical, and neuromodulation therapies is still limited, not completely understood, but encouraging [9]. Few papers (mostly case reports and case series) reported the outcomes of patients affected by RSE and SRSE treated with non-pharmacological therapies [10,11,12,13]. Focusing on neuromodulation, vagus nerve stimulation (VNS) is an approved treatment for drug-resistant epilepsy (DRE), but insufficient evidence supports VNS for the treatment of SRSE (27 class IV studies) [14]. A recent systematic review, including four previously published subjects of our series [10], found acute VNS implantation to be associated with RSE and SRSE cessation in 74% of cases. The mean duration of SE after implantation was 8 days, with a range of 3 to 84 days [15]. 

In this work, we present our findings of pediatric patients affected by RSE and SRSE treated with VNS in an acute setting and discuss the potential role of VNS as a promising neuromodulation therapy for this neurological emergency. 

## 2. Materials and Methods

Among all the children treated with VNS for drug-resistant epilepsy in San Gerardo Hospital (Monza) and in Padua University Hospital since 2012, we analyzed those implanted for RSE and SRSE. We collected a cohort of 7 consecutive pediatric patients treated with VNS by a single surgeon (AL) in Monza and Padua. All the patients were operated on with a standard technique and implanted with the Demipulse 103 or Sentiva 1000 device (Livanova PLC, London, UK) on the left vagus nerve, with the generator allocated in the subpectoral region [16]. A rapid titration up to 1 mA intensity in the first 24–36 h was started soon after implantation in all cases. We considered age at implantation, diagnoses, and electroclinical data collected through both clinical reports and EEG recordings before and after VNS implantation and at the last follow-up. We also reported stimulation parameters at the time of SE resolution for each patient (output current, frequency, pulse width, and duty cycle) and adverse events. The neurological outcome was assessed through the McHugh classification of seizure outcome for VNS [17,18]. 

## 3. Results

Patient 1: Female, 16 months at implant, with a diagnosis of left hemimegalencephaly, psycho-motor delay. She had been experiencing 90–100 focal seizures per day since she was 4 months old, developing focal RSE requiring pediatric intensive care unit (PICU) admission and treatment with multiple ASMs and anesthetics. VNS implant, conducted after 18 days of ineffective treatments, led to SRSE resolution after 4 days (1 mA, frequency 30 Hz, duty cycle (DC) 10%, and pulse width (PW) 500 us). No further SE relapse was recorded, but the child continued having 4–5 seizures/day. The stimulation parameters were gradually increased, and at the last follow-up, they were as follows: intensity 2 mA, frequency 30 Hz, PW 250 usec, ON Time 30 s, and OFF Time 3 min. At a 9-year follow-up, she exhibited 3–6 focal seizures per day (McHugh class IA), along with language delay, tetraparesis, and a reliance on gastrostomy feeding.

Patient 2: Male, 16 months old, with a diagnosis of non-ketotic hyperglycinemia with neonatal DRE. He first developed RSE at 5 months of age and second focal RSE at 16 m.o., during which he was implanted with VNS after 5 days. No other pharmacological interventions were carried out, and SRSE resolved after 5 days of VNS at 1 mA, 30 Hz, DC 10%, and PW 500 us. He remained seizure-free for 8 years (McHugh class IA), but he recently died from pneumonia and renal failure as complications of the rare genetic disorder. He suffered severe cognitive impairment, language delay, and tetraparesis.

Patient 3: Female, 17 m.o., with a diagnosis of microdeletion 1q43q44 syndrome with microcephaly and epilepsy. She first developed focal SE at 8 months and second RSE with bilateral diffusion at 16 months, requiring PICU admission. She clinically developed sub-continuous bilateral asymmetrical epileptic spasms, refractory to multiple ASMs and sedation. VNS at 1 mA, 30 Hz, DC 10%, and PW 250 us was implanted after 16 days, and then the spasms stopped after 36 h without any other associated treatment. At a 7-year follow-up, it was recorded that she never developed a new SE, but she recently experienced a few clusters of spasms per day (McHugh class IA), resistant to vigabatrin. She has a severe intellectual disability and is not able to walk.

Patient 4: Male, 7 months old, with a diagnosis of epilepsy of infancy with migrating focal seizures. He developed partial migrating motor SE at birth. He experienced repeated relapsing motor RSE, requiring PICU admission, and after 90 days, he was implanted without success (Mc Hugh class V). He developed SRSE and died a month later.

**Figure 1:**
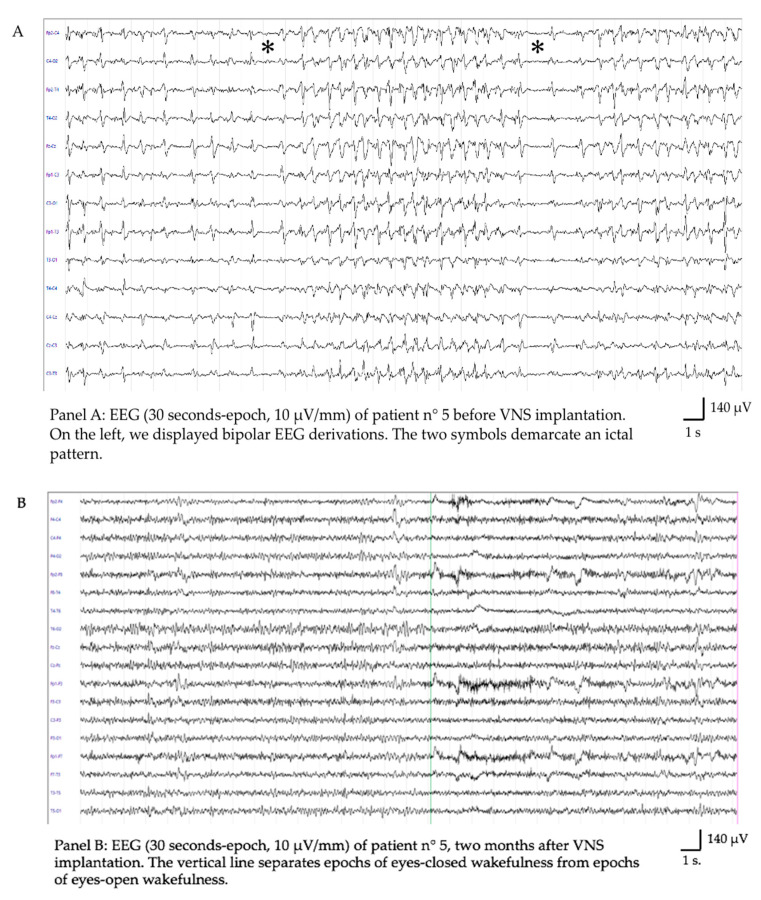

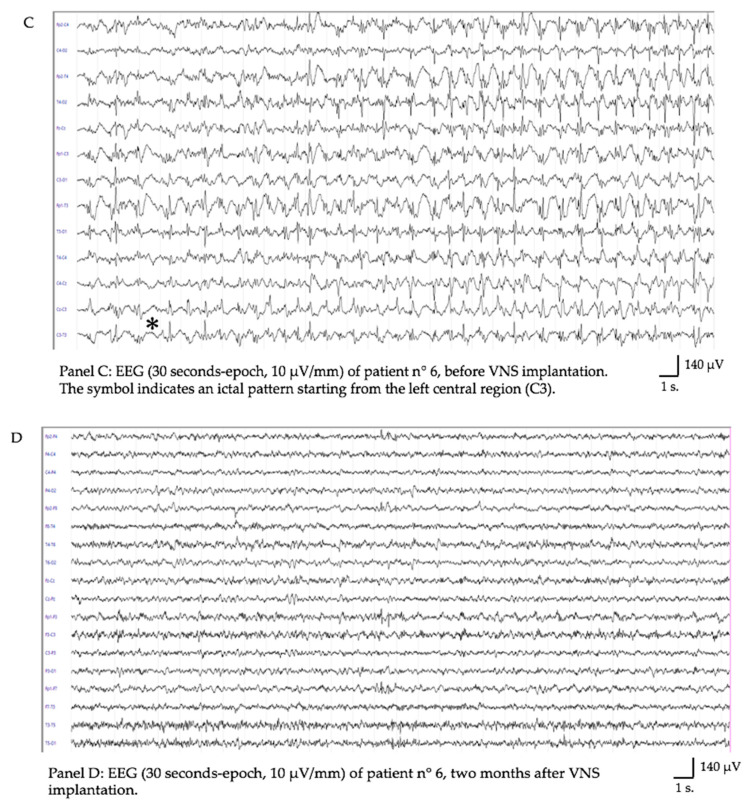
Two samples of electroencephalographic evolution following VNS implantation. Panels (**A**,**C**) demonstrate in two different subjects the persistence of status epilepticus despite the sequential treatment with anti-seizure medications, anesthetics, and immunomodulant therapies. Panels (**B**,**D**) display the interictal EEG of the same patients 2 months after VNS implantation.

Patient 5: Female, 14 years old, with no previous history of epilepsy. She developed a generalized myoclonic NORSE (new onset RSE) with a diagnosis of FIRES (febrile infection-related epilepsy syndrome). She was implanted during her PICU stay with VNS after 43 days of prolonged SRSE and treated with multiple ASMs, anesthetics, immunotherapy, and a ketogenic diet. VNS parameters were rapidly ramped up to 2,25 mA, 30 Hz, DC 16%, and PW 250 us. Twenty-six days after achieving the target stimulation parameters, off-label CBD was added (2.5 mg/kg/day, to be titrated every 5 days up to 12.5 mg/kg/day). Forty-eight hours later, the patient recovered from SE (Figure 1A), gaining a good clinical outcome, with only mild neuropsychological impairments (i.e., mild difficulty collecting and retelling recent memories) but with an exacerbation of prior psychiatric problems. She presented with mood deflection, anxiety, oppositional behavior, a sleep–wake cycle disturbance, and avoidant restrictive food intake disorder. Five months after VNS implantation, we were able to reduce the ASM burden by stopping gabapentin and topiramate; moreover, the intensity of stimulation was decreased from 2.25 to 1.75 mA. Her therapy currently consists of three ASMs (phenobarbital, lacosamide, and cannabidiol), and she was seizure-free at her 1-year follow-up (McHugh class IA). She talked properly and fluently, resumed homeschooling, and could walk with assistance.

Patient 6: Male, 6 years old, with no previous history of epilepsy. He developed NORSE/FIRES with a need for PICU admission and complex treatment with multiple ASMs, anesthetics, immunotherapy, and a ketogenic diet. He was implanted with VNS during his PICU stay after 26 days of RSE, with regression of SE after 28 days (Figure 1B). VNS parameters at the time were 2 mA, 20 Hz, DC 15%, and PW 250 us. Between the attainment of the targeted stimulation parameters and the resolution of status epileptic, two additional pharmacological therapies were introduced: ethosuximide (22 days following VNS implantation) and cenobamate (26 days post VNS implantation). At his last follow-up (one year), he had sporadic focal non-motor seizures (one per month) despite pharmacological therapy based on three ASMs (McHugh class IA). He developed some neuropsychological deficits and behavioral problems, such as disinhibition, mannerisms, and closing-in phenomena.

Patient 7: Female, 6 years old, with no previous history of epilepsy. She was admitted to the PICU with a diagnosis of NORSE/FIRES and treated with anesthetics, ASMs, immunotherapy, and a ketogenic diet without success. On day 25 after onset, she was implanted with VNS, but she is still in the PICU (4 months after onset) with a SRSE lately partially controlled by the induction of a burst-suppression pattern through sodium thiopental infusion (McHugh class IIIB).

Our results are summarized in Table 1. Overall, we achieved the resolution of SRSE in five out of seven patients in a mean time of 16 days (range 4–28 days); one patient died during SRSE, and another one died 8 years after SRSE due to other complications of his metabolic disease. The most recently implanted patient is still in PICU with partially controlled SRSE. At the last available follow-up, the five responders had a significant reduction of seizure burden without any relapse of SE (80–100% seizure reduction). One patient of the series experienced transient adverse events related to vagal stimulation, i.e., persistent coughing and a paradoxical tachycardia without other plausible causes; these symptoms were weaned with proper downregulation of VNS parameters.

## 4. Discussion

There are very few reports about RSE and SRSE treatment with VNS, and only the most recent ones have complete data about implant timing, stimulation parameters, and electroclinical responses [12,13,19,20]. The last systematic review by Dibué-Adjei et al. [15] found that 74% of RSE and SRSE in 38 acute VNS implantations in both adult and pediatric patients were successful in teminating the status. Extrapolating the results, taking into account the pediatric patients only, they report 84,6% of RSE resolution related to VNS.

In our small series of seven exclusively pediatric patients, we observed the resolution of SRSE in five patients implanted with VNS in the acute phase (71.4%), a rate similar to the one previously reported in the literature. It is important to highlight that in these severe and acute situations, we proceed with a rapid titration (in 24–36 h) differently from other kinds of drug-resistant epilepsy. There have been no SE relapses, and the seizure burden was significantly low, with at least 90% seizure reduction in all epileptic patients (McHugh class IA). To date, we had two non-responders: the youngest patient, a 7-month-old boy with epilepsy from infancy with migrating focal seizures, who died after a month, and a 6-year-old girl with FIRES who is still under PICU care with partially controlled SRSE through sodium thiopental infusion.

Regarding etiology, the first three responders had epileptic syndromes with structural, genetic, or metabolic etiology, while the last two responders were healthy children who had NORSE with clinical diagnoses of FIRES at onset. To the best of our knowledge, there are only a few other reports of NORSE/FIRES successfully treated in the acute phase with VNS [12,13]. We registered two different latencies of SE interruption: epileptic patients responded early after VNS implant (4 to 5 days), while patients with FIRES responded after 28 days.

In the first three patients who already had a diagnosis of epilepsy before the onset of RSE, no other post-VNS implantation interventions were carried out. Regarding patient five, twenty-six days after surgery, off-label CBD was added (2.5 mg/kg/day, to be titrated every 5 days up to 12.5 mg/kg/day). However, only 48 h after CBD introduction, status epilepticus weaned, and seizures stopped. It is improbable that the cessation of status epilepticus can be solely attributed to the administration of CBD, considering the brief time lapse and low dosage. The synergistic effect resulting from the combined application of CBD alongside VNS therapy presents an intriguing perspective. CBD, known for its capacity to reduce proinflammatory processes, seems to collaborate with VNS therapy. Research examining the anti-inflammatory actions associated with neuromodulation revealed a decline in neurotoxic substances following VNS implantation, coupled with an elevation of neuroprotective kynurenine metabolites [21]; this was accompanied by the normalization of cortisol levels and a reduction in interleukin-6 (IL-6) levels, observed specifically in individuals who responded positively to the treatment. Furthermore, contemporary investigations have unveiled that CBD demonstrates direct excitatory effects on vagal afferent neurons [22]; this observed excitation potentially elucidates a synergistic mechanism, contributing to an enhanced and facilitated neuromodulatory action.

In the case of patient six, between the attainment of the targeted stimulation parameters and the resolution of status epileptic, two additional pharmacological therapies were introduced: ethosuximide (22 days following VNS implantation) and cenobamate (26 days post VNS implantation). This scenario raises more doubts regarding the specific role of VNS in this context and questions the plausibility of a potential synergistic effect between these specific treatments. Notably, functional imaging studies have indicated the modulation of the thalamus by VNS [23,24], while ethosuximide primarily targets thalamic calcium channels. However, the decision-making process behind these medication choices was primarily guided by an anti-seizure strategy. Post VNS implantation, a gradual adjustment of sedative medications was feasible, resulting in oscillations between heightened alertness and unconscious states. Consistently with the observation of fragmented epileptiform discharges during periods of alertness and of gradual focalization of electrical patterns, the choice was made to administer a drug recognized for its efficacy in managing status epilepticus during sleep (ethosuximide) and to enhance a focal seizures therapy (cenobamate).

Moving from specific cases to broader observations, it appears that patients previously diagnosed with drug-resistant epilepsy exhibit more rapid responses to VNS therapy in case of status epilepticus, often without necessitating additional treatments. Conversely, in patients with FIRES, the response to VNS therapy takes longer, often requiring adjunctive treatments. Additionally, the natural course of FIRES indicates a resolution over several weeks to months. According to the most recent review by van Baalen in 2023, the median duration for resolution spans approximately 3 weeks, with a range extending from one day to 5 months [25]. However, our two responders showcased a resolution of status epilepticus within a similar timeline subsequent to VNS implantation (28 days) despite the application of different pharmacological treatments. This observation implies a potential overarching role of VNS therapy, possibly involving anti-inflammatory effects, reduction of cerebral excitability, and the modulation of electrical pattern synchronization, which appears to complement patient-specific treatments.

Another clinically relevant aspect is the potential benefit of VNS therapy on drug-resistant epilepsy following RSE/SRSE and, specifically, on the recurrence of status epilepticus. In our case series, no recurrence of SE was reported. A recent study [26] evaluated the efficacy of VNS through a follow-up of approximately 69 months in 125 patients from 14 to 44 years of age with drug-resistant epilepsy. In total, 56% of patients showed at least a 50% reduction in seizure frequency; however, one element that seems to predict which subjects will be non-responders is a history of SE prior to VNS implantation. On the other hand, 67% of patients with a history of SE showed no recurrence of SE; this suggests that in DRE, the reduction of seizure frequency and the prevention of the recurrence of SE are two distinct elements that are perhaps underpinned by two different mechanisms of action of the VNS. Overall, within the entire study population, the etiologies of epilepsy were distributed as follows: structural (45%), genetic (22%), unknown (20%), and infectious (13%). The etiology of epilepsy in cases with a history of status epilepticus (SE) prior to the VNS implantation is not explicitly documented in the aforementioned study. Another multicenter investigation focused on the recurrence of status epilepticus episodes following VNS implantation in adult patients with drug-resistant epilepsy [27]. Among the eight patients with a previous history of SE, a complete absence of new episodes was observed in 50% of cases. With respect to the etiology of epilepsy, it was found to be structural in three patients and unknown in another three, while two patients were diagnosed with autosomal dominant nocturnal frontal lobe epilepsy (ADNFLE). Notably, the four patients who exhibited a positive response included two with ADNFLE and two with unknown etiology.

Moving from drug-resistant epilepsy to NORSE/FIRES, of our two FIRES patients in the chronic phase, one is seizure-free, while the second tends to have clusters of seizures during infections but has not recurred SE. Overall, it is difficult to evaluate the safety and efficacy of VNS in the post-acute phase of NORSE/FIRES because of the paucity of the available data. A recent clinical review of FIRES in childhood [25] reported that in the chronic phase, seizures tend to occur in clusters, and they could evolve into status epilepticus in specific conditions, for example, during a febrile illness. In this perspective, the neuromodulatory and anti-inflammatory effects of VNS can play a role and should be considered, as stated in the 2022 International consensus recommendations for the management of NORSE [28,29]. We, therefore, believe it is necessary to expand the case series and have longer follow-ups in order to obtain more solid clinical data.

Concerning tolerability, tachycardia and coughing were the only adverse reactions reported in our series, and they were easily resolved by regulating the VNS output parameters. Usually, VNS causes bradycardia; however, the patient suffered frequent coughing and, at the same time interval, experienced tachycardia without underlying cardiac or systemic cause. We hence suggest tachycardia could be a rare adverse event related to stimulation because of the timing and the reversion with VNS downregulation.

Finally, from a neurophysiological point of view, more and more data are emerging from both animal and human models that provide clues on the possible antiepileptic mechanism of VNS [30]. The most interesting elements are a desynchronization in theta frequencies [31,32], a decrease in interictal epileptiform discharges [33,34], a decrease in cortical excitability with a non-linear trend compared to current output [35], and a global decrease in functional connectivity [36,37]. The reduction of interictal functional connectivity seems to play an important role both in predicting responders [38] and in constituting the main neurophysiological substrate of the therapeutic effect of VNS in drug-resistant epilepsies. However, some studies have shown the importance of modulating functional connectivity even during a seizure: when delivered within the appropriate time frame, VNS may decrease spatial synchronization [39].

The efficacy of VNS in RSE and SRSE could, therefore, be due to a reduction in temporal synchronization, spatial synchronization, and cortical excitability. All these features seem to have a non-linear correlation with the stimulation parameters (sometimes allowing us to hypothesize that response to VNS obeys an inverted U shape curve); consequently, not only the etiology underlying RSE but also the individual variability becomes fundamental. In this sense, more studies are warranted to improve protocols of implantation time, parameter titration, and monitoring of efficacy in seizure reduction or SE termination. Equally important, especially in terms of future perspectives and personalized medicine, is the identification of neurophysiological biomarkers that can discriminate responders from non-responders and optimize the stimulation parameters on the individual patient. Based on the above, we believe that quantitative indices of functional connectivity could be promising biomarkers.

We are fully aware of the significant limitations of this study, which is retrospective and includes a small sample size. The population consists only of pediatric patients and, for this reason, can be only partially matched with the series present in the literature. Other limitations are the timing of the VNS response, which is variable and differs between chronic epileptic patients and NORSE patients, and the difficult identification of the specific role of VNS in RSE/SRSE interruption among the several treatments used.

## 5. Conclusions

From a clinical perspective, our findings warrant contextualization within two distinct scenarios. In cases of RSE/SRSE occurring in patients already diagnosed with drug-resistant epilepsy, we suggest the early consideration of VNS therapy. This suggestion is based on the potential for prompt responses (these patients seem to exhibit more rapid responses to VNS therapy without necessitating additional treatments) and the aim to mitigate the pharmacological burden in patients already managing polytherapy. Regarding patients affected by NORSE/FIRES, the current International Consensus Recommendations for the Management of NORSE predominantly suggest the utilization of VNS therapy in the chronic phase, particularly for managing drug-resistant epilepsy following SRSE. Our case series also aligns with this recommendation since our patients had a good epileptological outcome without recurrence of status epilepticus. However, we propose that neuromodulation, with a rapid ramp-up of stimulation parameters, could also be contemplated during the acute phase. This consideration arises once primary and secondary treatment lines, such as anesthetics, immunomodulatory drugs, and the ketogenic diet, have proven ineffective or are poorly tolerated. We suggest, in these cases, a potential overarching role of VNS therapy, possibly involving anti-inflammatory effects, reduction of cerebral excitability, and the modulation of electrical pattern synchronization, which appears to complement patient-specific treatments. We, therefore, believe it is necessary to expand the case series and have longer follow-ups in order to obtain more solid clinical data.

## Figures and Tables

**Table 1:** Clinical characteristics, VNS details, and outcomes of the sample.

	Sex	Diagnosis	Age	Time Onset-to-implant (days)	RSE Stop	Latency to SE Resolution (days)	Stimulation Parameters (at SE Resolution)	Follow Up	McHugh Score	Level of Function	Outcome	Adverse Events due to VNS
**Pt1**	F	Left Hemimegalencephaly	16 mo	18	Yes	4	1 mA, 500 usec, 30 Hz, DC 10%	9 ys	IA	Unable to speak Tetraparesis	Alive	No
**Pt1**	M	NonKetotic Hyperglycinemia	16 mo	5	Yes	5	1 mA, 500 usec, 30 Hz, DC 10%	8 ys	IA	n/a	Deceased for metabolic cause	No
**Pt3**	F	Microdeletion of 1q43q44	17 mo	16	Yes	3	1 mA, 250 usec, 30 Hz, DC 10%	7 ys	IA	Unable to walk Profound mental retardation	Alive	No
**Pt4**	M	Migrating Epilepsy	7 mo	90	No	n/a	n/a	1 mo	V	n/a	Deceased during RSE	No
**Pt5**	F	FIRES	14 ys	43	Yes	28	2.25 mA, 250 usec, 30 Hz, DC 16%	1 y	IA	Mild neuropsych impairment	Alive	Tachycardia Coughing
**Pt6**	M	FIRES	6 ys	26	Yes	28	2.00 mA, 250 usec, 20 Hz, DC 15%	1 y	IA	Behavioral problems	Alive	No
**Pt7**	F	FIRES	6 ys	25	No	n/a	n/a	4 mo	IIIB	n/a	Alive	No

Legend: DC: duty cycle; F: female; FIRES: febrile infection-related epilepsy syndrome; M: male; mo: months; n/a: not available; SE: status epilepticus; VNS: vagal nerve stimulation; ys: years.

## Data Availability

The data presented in this study are available on request from the corresponding authors. The data are not publicly available due to privacy restrictions.
